# Methods of Assessment of the Welfare of Shelter Cats: A Review

**DOI:** 10.3390/ani10091527

**Published:** 2020-08-28

**Authors:** Veronika Vojtkovská, Eva Voslářová, Vladimír Večerek

**Affiliations:** Department of Animal Protection and Welfare and Veterinary Public Health, Faculty of Veterinary Hygiene and Ecology, University of Veterinary and Pharmaceutical Sciences Brno, 612 42 Brno, Czech Republic; voslarovae@vfu.cz (E.V.); vecerekv@vfu.cz (V.V.)

**Keywords:** welfare, animal-based measures, assessment, cats, shelters

## Abstract

**Simple Summary:**

The welfare of animals in shelters draws the attention of both the scientific and general public. It is possible to assess the well-being of cats in shelters using tools that are based on indicators used to reveal problematic aspects of welfare. This review aims to provide an insight into available methods of assessment of the welfare of cats in shelters with an emphasis on behavioural, physiological and health indicators.

**Abstract:**

At any moment, there are millions of cats housed in foster care facilities for abandoned and stray animals for various reasons worldwide. Care, management and regulation among these facilities differ. Moreover, shelters can never substitute the full comfort of a good home for the animal, and the welfare of cats in shelters is a subject of discussion in many respects. Cats are animals sensitive to changes; for most of them, placement in a shelter is a stressful experience because of changes in routine, environment and the presence of other animals. Stress is reflected in changes in behaviour, causes fluctuations in physiological values and disrupts the immune system, which is a predisposition to the development or reactivation of disease. Evaluation of the presence and intensity of negative impacts is possible through the use of evaluation tools based on indicators that help set the environment and management of keeping so as to disrupt the quality of life as little as possible. Although a comprehensive and valid welfare tool that would evaluate animal-based and at the same time resource-based (or management-based) indicators of cats in shelters is not currently available, it is possible to use partial evaluation of individual welfare indicators to assess welfare. This review aims to provide the readers with an insight into current options of assessment of the welfare of cats in shelters with an emphasis on behavioural, physiological and health indicators with an application in both practical and scientific contexts.

## 1. Introduction

In recent years, cats have become the most popular pet animals in Western Europe and the United States. Their number in the European Union reached over 103 million [1] and in the United States over 94 million [2]. The result is an increased focus on issues dealing with their well-being and behaviour [3,4].

At any moment, there are millions of cats housed in shelters around the world [5]. For example, approximately 3.2 million cats enter shelters in the United States every year [6]. In Canadian shelters, 600,000 cats were housed in 2011 [7]. In 2009, 131,070 cats entered shelters in the UK [8], 33,719 cats were housed in shelters in Spain in 2018 [9] and about 7400 cats are housed in shelters in Sweden annually [10]. The traditional view that animals with unrestricted movement pose a risk to public health and safety, and that the solution is euthanizing them, has already been partially overcome. In most Western countries, the approach to solving the problem has changed over the last 30 to 40 years; non-governmental organisations and in some countries, public authorities have invested in programs to increase the number of successful adoptions to minimise the need for animal euthanasia [11]. In order to eliminate euthanasia of animals in shelters, some countries (e.g., Czech Republic, Italy, Sao Paulo, Austria, India, Taiwan, Germany, Costa Rica) [12] have adopted the so-called no-kill policy. In these countries, shelter cats can only be euthanised for medical reasons. In other countries, on the other hand, the shelters themselves determine the functioning policy; for instance, some no-kill shelters refuse to accept animals that are sick, elderly or have unacceptable behaviour because they are bad candidates for adoption. However, this type of restrictive policy can also lead to worsening of welfare in an unwanted group of animals and overcrowding in shelters that have open admission policies [13]. All facilities providing temporary care should aim to reduce the animal’s stay there to a minimum. A problem can arise if the no-kill policy orders facilities to accept all animals and keep them in a shelter until they are adopted by a new owner, returned to the original owner, die of natural causes or are put down for health-related or behavioural reasons. An animal that no one is interested in (often an older animal or an animal with a disability) can therefore legally stay in a shelter for a very long time. However, shelters are generally not designed as facilities that would be able to replace a new home in the long run; the animal generally has limited living space and access to resources, which it often shares with other animals with various medical histories. Since a multitude of facilities does not have a sufficient amount of staff at their disposal, only a minimum of time is reserved for the care of each animal; Ammons [14] reports an average of 15 min per day per animal.

The common goal of most facilities is to temporarily provide the animals with a suitable space that takes into account nutritional, housing, health care and human contact requirements. The quality of care is a critical aspect of cat welfare in many facilities [15]. Various factors of shelter environment can be stressful for cats (different care routine, lack of a familiar/bonded caretaker, veterinary treatments, increased infectious pressure, the presence of other animals, inadequacy in terms of space or poor environment and overall lack of control over the environment) [16,17,18,19,20]. The intensity and number of negative factors may reflect the condition of the shelter; in many countries, the system for dealing with unwanted animals is complex, lacks comprehensiveness and the cooperation between the state (or state facilities) and private facilities is not functional; likewise, the level of supervision in various types of facilities is different, sometimes even completely absent. The standard of maintenance and care of animals can vary significantly across facilities, as can their funding. Insufficient funding may be a factor causing a number of other potential animal welfare problems [13].

Animal welfare can generally be assessed from three different perspectives [21]. The first defines well-being through a biological point of view—the welfare of the animal is preserved if there is no deterioration in health and reproductive ability. The second view assumes that well-being depends on the ability to engage in natural behaviour. Although this approach is traditional, today there is a tendency to attribute feelings and emotions to animals; trying to understand mental processes creates another perspective on welfare. The third view of the welfare concept, applied in the manuscript, involves the assumption that animals are sentient beings capable of experiencing positive and negative emotions [22]. Because the subjective state of well-being is the result of the mental processes which are intrinsic to the animal in which they take place and invisible to the eye, its assessment is difficult. The fact that animal welfare is based on the subjective states perceived by the animal prevents us from its accurate assessment in spite of utilising any available methods. While current evaluation methods do not possess the capability (or this capability is limited) to provide direct insight into the mental state of the animal, at least the indirect methods are available [23].

The development of the first protocols for the practical assessment of welfare began in farm animals. Efforts to evaluate welfare first led to the creation of some indicators of well-being; these focused on key aspects—health, behaviour, housing and management of keeping [22]. It is now customary to divide these indicators into two groups—a group of indicators, which directly relates to the evaluated animals (indicators of behaviours, health and physiological markers of stress [23]) and a group concerning the resources of the environment in which the animal lives. It is appropriate to assess the well-being of the animal in terms of comparing the provided living conditions with the optimal environment, and in terms of the strategies that the animal must apply to cope with the difficulties and successfully adapt to the conditions [23]. The Welfare Quality project [24], focusing on cattle, pigs and poultry, proposed a scientifically valid welfare assessment system and its four principles (good feeding, good housing, good health, appropriate behaviour) became a key basis in designing tools and evaluating the welfare of two other categories of animals—laboratory animals and companion animals. While in the past the general interest was concentrated on livestock (with the intensification of agriculture linked to the emergence of large-scale livestock farming, it was natural for questions pointing to the deteriorating living conditions of farmed animals to arise), attention has now also shifted to species that seemed previously least affected.

Unlike in cats, tools that assess the well-being of dogs have been the subject of several studies [25,26,27,28,29,30,31,32,33,34,35,36]. In addition, evaluation tools have been developed to assess the behaviour of dogs in terms of its prediction in the home environment of the new owner. The tests are mainly aimed at detecting aggression [37,38,39,40,41,42,43,44,45]. The tools have been criticised because some authors [46,47] consider them to be scientifically insufficiently validated to decide on the future of the animal (often, based on testing, the shelter decides to move the animal to another facility or euthanise it because of unacceptable behaviour or low adoption potential). Shelters in none of the European Union countries use an officially validated tool to assess the behaviour of dogs in shelters [48].

Behavioural tests have also been developed in shelter cats [49]; their main aim, once admitted to a shelter, is to discriminate socialised animals from unsocialised ones in order to preserve their welfare. Temperament tests form another group of tools used in cat shelters; the goal is to assess the cat’s temperament and find a compatible new owner [50].

However, the situation in evaluating the welfare of cats in shelters is different compared to dogs—at present, there is no comprehensive well-being evaluation tool usable in cat shelters. Efforts to improve animal welfare are noticeable, however, e.g., within the European Union, a number of member states have not laid down a legal obligation regarding any specific procedures to be performed after the animal is admitted to a shelter or during its stay in a shelter [48]. Since there is no comprehensive tool for evaluating the welfare of cats in shelters, it is desirable to consider currently available tools that enable to assess individual aspects of welfare, i.e., to use the indicators that have been developed to evaluate the welfare of cats under specific conditions. The aim of the review was to provide the readers with an insight into welfare assessment tools, whose criteria may form the basis for the development of a complex, more comprehensive and valid tool evaluating the welfare of cats in shelters and summarise additional options of assessment of welfare of cats in shelters with an emphasis on behavioural, physiological and health indicators.

## 2. Assessment of Cats’ Welfare

### 2.1. General Aspects of Welfare Assessment

Welfare or Quality of Life (QoL) are interrelated concepts and are often interchanged; according to some authors [51,52,53,54] their definitions are identical. While the welfare concept has been presented only for the last few decades (one of the first definitions of welfare was introduced by Broom in 1986 [55]), the origin of the term ‘Quality of Life’ dates back to the days of Plato and Aristotle, who used the term to examine the conditions necessary to live a ‘good’ life [56]. The principle of the original concept, which became widely accepted, was to assess the quality of human life in terms of health aspects [57]. The term QoL was later introduced into veterinary medicine; the concept began to apply when deciding between treatment and euthanasia of an animal in veterinary clinics [58,59].

Although there are several approaches to understanding the focus of the concept of QoL, Taylor and Mills [57] have provided a general definition of QoL applicable in various contexts. According to the authors, QoL can be defined as an individual state that an animal perceives at a given moment in its existence; the condition should include a balance between the positive and negative attributes of life. Cummins [60] adds that well-being is the result of many factors; it is a multidimensional construct.

While tools for assessing farm and laboratory animal welfare are based largely on five freedoms [61] (freedom from hunger, thirst, pain, injury, illness, discomfort, fear and anxiety and freedom to express normal behaviour), the evaluation of companion animals is mostly focused on the use of health and behavioural parameters that can provide insight into the animal’s internal state and its ability to cope with adverse conditions [62]. Stress (especially distress) has an impact on the quality of life of animals [54]. In addition to the evaluation of behaviour and health, it is possible to evaluate the worsened welfare or the ongoing stress response by using physiological markers of stress [23].

It is challenging to select indicators that, in turn, represent positive emotions and good living conditions, i.e., indicators or conditions that reflect a good quality of life. The proposed indicators of positive emotions reflect the positive cognitive attitude of the individual; these are behaviours such as play, exploration, vocalisation, social behaviour and grooming [63,64]. However, some of these indicators have not been fully validated in order to be usable in practice. In developing welfare assessment tools, reliability and validity are key to assessing whether the resulting concept actually measures what it was designed to [65]. Reliability is expressed as a measure of repeatability and consistency [66] and can be measured within a single observer (intra-observer) and between multiple observers (inter-observer), within a test (test-retest) and between components which are the subjects of the test (internal–consistency) [67]. It does not mean that a tool that was previously validated in the original language is automatically valid when its language is changed or when it is used in other cultural contexts [68,69]. Before assessing reliability and validity, the purpose and standardisation of evaluation need to be addressed [67], taking into account practicality and executability [65]. There is a risk that measurements that take a long time or are too complex will not be performed correctly [67] or will not be used at all.

Some of the practical problems in assessing welfare or QoL are similar to those that arise in assessing people with communication and cognitive impairments. Like them, animals cannot provide their own self-assessment [70,71,72]; therefore, questionnaire methods such as those common in human medicine cannot be used to assess their quality of life [73]. Questionnaires can be used as comprehensive tools containing objective records [74] on nutrition, level of activity, environment, social interactions and behaviour when filled out by a person closely related to the animal [75], i.e., the caregiver of the sheltered cat. However, the disadvantage of most animal-based measurements is their subjectivity [76]. The caregiver is required to interpret the animal’s expressions correctly, which is a problem if shelter staff are not sufficiently educated and trained—testing can yield anthropomorphised, biased findings. Results can also be influenced by the principles and feelings of the individual who evaluates the animal [75].

Traditionally, quality of life assessment can focus not only on the assessment of the animal itself but also on the assessment of resources [77] and management (thus a combination of several indicators is used for evaluation), as these remain constant and can be measured objectively (e.g., assessment of appropriate housing, nutrition and the human factor). An isolated assessment of resources is insufficient because it does not provide a real picture of the level of welfare, but only indicates the risk of its deterioration; although optimal management may be set up in the shelter facility and the animal may have all the necessary resources at its disposal, this does not automatically mean that it is healthy and provided with a high standard of welfare. A comprehensive welfare assessment tool should, therefore, be based primarily on the parameters of the animal, supplemented by other parameters. In some studies, the approach of assessing quality of life only from the point of view of health is maintained [25]. If welfare assessment is set up properly, it takes into account feedback on changes that affect the animal’s welfare and allows for appropriate course of action to be taken thereon [78].

### 2.2. Compehensive Welfare Assessment Protocols

The majority of tools assessing the quality of life that are available today tend to focus on evaluating the lives of cats as patients with certain types of health problems, such as diabetes mellitus [71], cancer (see [79] for the review of current methods of QoL assessment in cats receiving chemotherapy), degenerative joint disease [80,81], heart disease [82,83], skin problems [84] or chronic disease in general [85]. These tools cannot be used for the evaluation of healthy cats, as they consist of disease-specific parameters. In addition to specifically targeted tools, efforts have been made to create more general tools that can be used in a variety of situations, facilities and contexts. Their purpose is to define and differentiate normal behaviour and health and their deviations that an individual exhibits. Some of these comprehensive tools (modified Karnofsky score, CHEW [Cat HEalth and Wellbeing] tool, owner completed measure of feline QoL, CatQoL tool, the AWAG software, Shelter Quality and the shelter dog QoL evaluation tool) could be considered for shelter cats’ welfare assessment after some adjustments (these tools were developed for the use in different context, so they are not able to cover the full range of evaluation criteria requirements emerging from the shelter environment) or their principles could be used when creating a new assessment protocol.

A QoL tool that can be used to assess aspects of cat health and well-being without specific health problems is the so-called Karnofsky rating scale, modified by Hartmann and Kuffer in 1998 [86]. It was originally designed to determine disease progression, treatment effects and the ability to perform normal daily activities in human cancer and HIV patients [87]. The Karnofsky Score (KS) modified for feline patients consists of two parts: the first part is the score given to the cat by the veterinarian (clinician’s score) after assessing its state of health and body condition; the second part consists of a questionnaire containing questions about behaviours, which are answered by the owner [86]. A high degree of correlation was found between the results of individual observers when assessing behaviours [88]. Lower inter-observer reliability for clinician’s score was found, requiring additional modifications; a standardised, detailed approach with appropriate criteria combined with more specific training on how to use the scale may improve inter-observer consensus and consistency. The score obtained from questionnaire seems reliable, so it may serve as a reliable tool in the assessment of QoL [88]. Although the KS assessment method is not primarily designed for cats in shelters, it is one of the few tools that can be used in this regard due to its relative non-specificity. It should be mentioned that the protocol could only be used for animals that have been in a shelter for a long time; the caregiver completing the questionnaire must be able to correctly estimate the level of reported activities of the cat.

The need to find a comprehensive but especially reliable tool for assessing mainly the clinical aspects of healthy cats also resulted in a study conducted by Freeman et al. [89], a group of American scientists. They first defined the attributes that cat owners consider relevant in health assessment; then they tried to create an evaluation tool (CHEW [Cat HEalth and Wellbeing]) based on these attributes. Parameters that are part of the assessment include easily determinable, animal-oriented items such as play, mood, energy, appetite, body condition and coat condition. The experiment resulted in the establishment of eight domains containing 33 assessment items. The authors mention that the final tool showed good validity, was able to discriminate overall health status, demonstrated good internal and test–retest reliability and is usable across various age categories of cats [89], which is an important aspect for its use in shelters, as they usually house cats in various age categories. On the other hand, its use in shelter environment has not been tested. However, because the tool is based on two types of animal-based indicators (it involves behavioural and health indicators), its use as a comprehensive tool for assessing quality of life of shelter cats could be considered.

A similar procedure (where its authors let the owners of cats determine, what behaviour they considered normal in the animals) was employed in tool creation by Tatlock et al. [90] as well—23 evaluation items were developed in 15 areas—general health, interaction with humans, vocalisation, pain, sleeping behaviour, appearance, interaction with surroundings, toilet habits, gastrointestinal symptoms, hydration, appetite, weight loss, grooming, general happiness and physical activity level. Authors reported strong internal consistency and test–retest reliability for all domains, indicating good reliability. In QoL tools, it is customary for each rating item to have the same weight; however, it should be noted that the importance and impact of the items included on quality of life may vary, which should be taken into account when assigning weight and calculating the overall QoL score.

In contrast to the aforementioned studies, in addition to animal-based indicators (general state of health, food intake, behaviour), the QoL tool (CatQol) proposed by Bijsmans et al. [91] takes into account management-based indicators and also the principle of evaluating each item included in the tool according to the frequency or severity with which it affected the cat’s life. The CatQoL questionnaire was validated by taking all aspects of the psychometric analysis into account and assessing each question individually. The disadvantage of the questionnaire is that it contains questions on cat’s experience during the previous week. Thus, a cat could not be evaluated sooner than after 7 days spent in a shelter. In addition, the authors mention that the result obtained reflects the nature of the relationship between the owner and the cat to a certain extent. Adamelli et al. [92], who analysed the relationship between a person and a cat with an emphasis on quality of life in 62 animals, concluded that the QoL of a cat may be more influenced by the characteristics of the owner than the characteristics of the cat. QoL was rated low, moderate or high in this study using four questionnaires that analysed care, behaviour and characteristics of the cat and the owner, a simple physical examination and Lexington’s attachment to pets scale (LAPS) test. The LAPS test, developed by Johnson, Garrity and Stalones in 1992 [93], is probably the most widely used questionnaire for assessing the emotional bond between humans and animals [94].

Although the described tools have been tested for future use by cat owners (in the home environment of cats), they represent at least a sketch or insight into the criteria that could be evaluated in the case of shelter cats. Also, other tools that have been developed to be used in various animal species and contexts could be considered for cats in shelters. One such tool is The Animal Welfare Grid (AWAG) software, which was originally designed to monitor animal welfare in research institutions (originally used in primates) [95]. The AWAG draws attention to the temporal component of any suffering, reflecting the cumulative lifetime experience of an individual. The system measures criteria covering four different parameters that impact animal welfare—health, psychological well-being, environmental quality and clinical and management procedural events. Within each parameter, a number of indicators are evaluated on a scale of 1 to 10; a score of one indicates the lowest impact on welfare, whereas a score of 10 represents the highest impact on welfare. For each parameter, the software calculates the mean score, which is used to create polygon; the total area of the polygon presents the cumulative welfare score of an animal at a particular moment [95]. The AWAG tool can be used either predictively or retrospectively, including monitoring specific events or procedures affecting an animal or group of animals. The advantage is that software is freely available on the project website (https://github.com/PublicHealthEngland/animal-welfare-assessment-grid/wiki) and that it is transferrable and can be used to manage the welfare of a wide range of species in a variety of settings [78]. Some studies have been conducted using the AWAG, however no study has described its use in a shelter environment, therefore, no information on possible problems in this context is available. A potential problem with applicability in a shelter could be the evaluation of indicators itself. The scale contains 10 evaluation points, i.e., it requires ability of the evaluator to distinguish subtle changes and deviations between scores. This could be an issue in behavioural assessment of shelter cats; shelter staff usually do not have information on temperament of admitted cats, so distinguishing subtle changes in their behaviour could be challenging.

The basic four principles (good feeding, good housing, good health and appropriate behaviour) used in the protocol evaluating the welfare of dogs in shelters [34] have general application in the assessment of welfare and are therefore applicable to almost any species of animal; they could therefore also be applied when assessing the welfare of cats in shelters. However, the individual evaluation criteria, of course, have to be adapted to the conditions in the cat shelters and to the cats themselves—the biological and ethological aspects of the cats should be taken into account in the evaluation criteria. The Shelter Quality protocol for evaluation of welfare of dogs that are older than 6 months and have been housed in a shelter longer than 2 months, developed in 2016 by the Italian Instituto Zooprofilattico Sperimentale dell’Abruzzo e del Molise, follows the criteria of reliability, validity and feasibility [57]. It is a tool that uses direct observation of animal responses while identifying important aspects of the environment and management to detect welfare risks [34]. Observations are made at the level of the shelter, the cage and the animal. In developing the evaluation criteria, the authors were inspired by the approach of the Welfare Quality Consortium [24], which developed protocols for the evaluation of livestock welfare [96].

Other principles have been incorporated by Kiddie and Collins [32] into the shelter dog QoL evaluation tool—unlike the animal-based, resource-based and management-based approaches applied in Shelter Quality, the QoL evaluation tool focuses only on animal assessment; the tool does not take into account environmental assessment and does not look at the management of the facility. The authors first compiled a list of behaviours that were associated with stress and behaviours that were associated with positive emotions according to literature and the experience of the shelter staff. The evaluation itself consists of 5 parts—3 parts require observation of the dog’s behaviour from a distance, in which positive and negative behaviours are recorded, the 4th part evaluates the dog’s reaction to a person during an interaction. In the last part, three parameters are evaluated by the observer—body condition—according to a 9-point scale, the presence of scurf and eye discharge. Each evaluation item can receive a score of 1 or 0, depending on whether or not the occurrence of the observed trait or behaviour is recorded. Kiddie and Northrop [97] launched a project called ‘Assessing the quality of life of kennelled cats’, which aims to create a similar rating system for cats in shelters. The final tool has not yet been published.

## 3. Deeper Insight into the Options of Evaluating Animal-Based Indicators of Cat Welfare in Shelters

In this section, we discuss the options of assessing three categories of animal-based indicators—behavioural (Section 3.1), physiological (Section 3.2) and health (Section 3.3), which can be used to evaluate the welfare of shelter cats in a practical and scientific context.

### 3.1. Options of Evaluation of Behaviours of Cats in Shelters, Taking into Account Existing Methods of Stress Assessment

For welfare assessment, it is important to select behavioural variables relevant to the studied species as indicators and to take into account its evolutionary history. The domestic cat evolved into a carnivore with a solitary lifestyle, so the development of exaggerated behaviour was not necessary. Assessing their well-being based on behaviour may seem all the more challenging [4,98]. In cats, some degree of variability in behavioural responses to stressful stimuli was found, which is probably influenced by temperament and personality; even its inheritance is presumed [99].

In this section, we discuss the behavioural indicators that indicate impaired welfare of cats in shelters and methods that can be used in practical as well as scientific evaluation of behavioural responses of cats to stress (Cat-Stress-Score assessment) and methods that are used to discriminate socialised and unsocialised cats (approach tests). In terms of the applicability of approach tests, we discuss tests usable in scientific (Cat-Approach-Test, Human-Approach-Test) and practical (Feline Spectrum Assessment) contexts.

In general, behavioural assessments can be perceived as subjective (namely considering the possibly subjective interpretation of observed behaviours by the rater) and are not always accepted as a valid indication of stress unless they are accompanied by the results of physiological measurements. The combination of physiological and behavioural data may provide credible evidence of the presence of stress [100], but there are cases, when this statement is not valid (e.g., Cat-Stress-Score does not correlate with the level of cortisol). However, shelter staff usually do not have sufficient resources to analyse physiological data, so it is useful to have an easy-to-use tool for assessing behaviour [101].

#### 3.1.1. Behavioural Indicators of Deteriorated Welfare of Shelter Cats

Cats could find particular aspects of the shelter environment stressful, such as confinement, the proximity of other unfamiliar cats, handling by caregivers and changes in routine [15,76,98,102]. On the other hand, a recent study by Vitale and Udell [103] found that shelter cats seek out human interaction (more than pet cats) so lack of human proximity may also be a stressor. Cats respond to the presence of humans and prefer contacting with them with toys [104].

Behavioural changes form the body’s primary response to stress, serve as an adaptation mechanism and reflect the animal’s internal state [105]. Stress is a common aspect of life and is experienced by all living beings [106]. A problem arises if the amount of stress that an animal is experiencing crosses a certain line and becomes undesirable, and the coping strategies to handle stress have been exhausted [107].

Although it has been found that the level of stress in sheltered cats decreases as they spend more time in the shelter [108] and that most cats adjust to the shelter environment within 2 to 5 weeks [109,110], some cats are unable to get used to it at all and thus remain in a state of chronic stress for a long time [105]. Gouveia et al. [111] studied the differences in the behaviour of cats kept in shelters in groups with various densities and sex ratios for various lengths of time. Cats that stayed in the shelter for a long time were less active and more involved in conflict situations.

The presence of stress in cats is more often manifested by inactivity with inhibition of natural behaviour (reduced food intake, insufficient coat care, reduced frequency of playful behaviour [5,112,113], persistent vigilance [15], agonistic behaviour [17] and the need to hide [15]) rather than any active manifestation of abnormal behaviour [4,99]. The aforementioned indicators of good and impaired welfare, along with some others, are summarised in Table 1.

The level of stress is affected by the quality of housing; the inability to show the natural range of activities for a longer period of time can lead to stress, which applies especially to individually housed cats in cage housing [130], often poor in enrichment, which is often used in shelters e.g., in the United States [131,132] or in general within the quarantine. The level of stress can be partially regulated by providing an undisturbed, dark place where the cat can hide [133,134], which, of course, does not address the overall lack of stimuli and space to engage in natural active movement. On the other hand, no difference was found in the stress responses of cats when comparing the amount of space provided per cat (1 m^2^, 2 m^2^, 4 m^2^) when the resources were the same [135]. This may indicate that it is not the size of the cage but its capacity to be utilised that has an impact. Group housing, chosen by a number of shelters because of lack of space, on the other hand, is not entirely satisfactory from the point of view of infectivity (as there is a constant change in the composition of groups) and of social structure—in nature, cats form groups only under certain conditions; one of them is the sufficient availability of resources. In addition, cat colonies are usually comprised of females and their offspring [136]. Grouping in shelters is therefore generally problematic when mutually unfamiliar and unrelated cats are housed together [137]. Some individuals in a group may prevent others from accessing resources due to their territoriality. In addition, cats have been found to be able to form a linear rank order when living in group; higher-ranking cats tended to gain weight, whereas lower-ranking cats tended to lose weight [138]. Escaping or avoiding another cat may be a problem, as it may not be possible in a confined environment. On the other hand, the advantage of group housing is an environment that usually provides cats with more enrichment. Cats have more space available for movement and more control over the use of their environment [135]. Uetake et al. [139] reported lower levels of activity in individual cage housing as opposed to group housing. This finding is in contrast to the findings of Guveia et al. [111]—cats that were housed in groups with a high number of individuals were found to generally show lower levels of activity [111]. There may be several reasons why the findings are inconsistent. Cats in any housing system are exposed to stress, but it comes from a variety of sources, and cats also have different options for coping with stress. As density is often related to stress, key information such as the total number of cats and the spatial characteristics of the room are often missing. Another complication when comparing cats from group and individual housing is the fact that the groups are not equal to each other and the studies do not take into account the characteristics of individual cats and the treatment of each group at the shelter [140]. Suchak and Lamica [140] attempted to address this issue by creating a retrospective matched cohort of cats in two housing systems. Cats housed in groups were matched with cats housed individually with the same characteristics (age, breed, sex, size and coat colour). Authors found no difference in cats’ experience while at the shelter. However, in this study authors sought to compare individually and group housed cats based on measures related to their management in the shelter and outcome; behavioural measures were not assessed. The discussion about which type of housing is less stressful is still ongoing and with ambiguous results [140,141].

#### 3.1.2. Methods Evaluating Shelter Cats’ Behavioural Responses to Stress (Cat-Stress-Score and Approach Tests)

The first scale developed to assess behavioural responses to stress was The Global Assessment Score, designed by Sandra McCune in 1992 [98]. It contained a description of 10 levels of stress in cats [98], the number of which was later reduced to 7 by Kessler and Turner in 1997 [76]; they used it in their study to compare cats housed in a shelter individually, in pairs and in groups. The scale was named ‘Cat-Stress-Score’ (CSS) and was well received by a wide range of professionals. It is currently a relatively simple and at the same time the most commonly used method for assessing behaviours of stress in cats [135,142]. The scoring scale consists of 7 grades, from the state of ‘total relaxation’ (score 1) to the state of ‘extreme stress’ (score 7). The resulting grade is obtained from a brief observation of the cat’s posture and behaviour [76] described in the ethogram developed by the British Cat Behaviour Working Group [143]. The authors suggest that a score below 3 is still acceptable because it represents only the basic level of stress present in any living animal. An increase above this level means a response to an acute stressor and does not present a problem as long as the score does not remain the same over time [109]. They also point out that the scale can be used in all housing systems except those in which the temperature does not reach at least 15 °C. At lower temperatures, animals naturally do not show a relaxed attitude, which could lead to skewed results [109]. The use appears to be reliable—McCune [98] mentions 100% repeatability among observers, Kessler and Turner [76] reported a 90% match among observers who received training in the use of the scale and a 75% match among untrained shelter staff. However, more research should be performed on how the CSS scale could be reconstructed for increasing its validity and reliability.

The disadvantage of the method is that the evaluation is based on a very short assessment period, is subjective [144] and the results may be influenced to some extent by the presence of some factors (e.g., age, sex, neuter status). Another significant disadvantage of the method is that the score does not correlate with the level of cortisol [101,142]. No correlation was found in the combination of evaluating the behaviour using the CSS scale and determining the ratio of cortisol to creatinine in cat urine; nor was it found when comparing the CSS test result with cortisol metabolites in the faeces [101,142]. The authors explained the absence of correlation by the fact that the ratio of cortisol to creatinine in urine was cumulative, while the behavioural score was dependent on environmental conditions at the time of assessment.

It is not clear whether the intervals between scores are equivalent (i.e., whether, for example, a change from score 3 to score 2 presents the same level of stress reduction as, for example, a change from score 6 to score 5) and also whether the use of intermediate stages in assessment is possible (when the cat exhibits behavioural elements of two separate grades at the same time) [145,146]. Decreased cat activity and suppressed natural behaviours that may persist for long periods of time and are a sign of stress may be misinterpreted by an untrained evaluator as calm and contented [105]. In order to improve the sensitivity to detect subtle variations in stress levels, Bradshaw et al. [102] added half steps between scores 2–4. On the contrary, cats that have impulsive personality achieve higher scores in certain aspects of the scale, which does not automatically mean that they are extremely stressed. If possible, because of the individuality of each cat, it is more appropriate to carry out the assessment at different times during the day or over the course of several days [50]. Observers may also misjudge manifestations of oestrus resulting in increased scores; an example is an increased rate of vocalisation [147].

The CSS scale has been used to assess cat stress in shelters in a number of studies [5,145,146,148]. It was found the score was highest in cats in the morning, and that the tool was not suitable for detecting pretended sleep and overly excessive stress levels [101]. In a study by Broadley et al. [144], the scale was used to compare the behaviours of cats (originating from multi-cat households and households with only one cat) after they were placed in separate cages in a shelter. Cats that came from households without additional cats could experience higher levels of stress in the first days in the shelter than cats that had previously been accustomed to the presence of other individuals, in spite of limited direct visual contact with other cats. Kry and Casey [133] used a scale to examine the effect of enrichment of individual housing in shelter using a BC SPCA Hide & Perch^TM^ box to achieve a lower score for cats for which this enrichment was available.

Other tests to assess behavioural responses are approach tests. These are used to evaluate responses of cats to unknown stimuli (unfamiliar humans or cats) approaching the cat to determine whether a cat is socialised or not. The individuality of a cat and socialisation are the most important factors influencing a cat’s behaviour toward humans and new situations in general [99,109,149,150,151]. The socialisation period begins in cats at the age of 2 weeks and ends around 7 weeks of age. The experience of kittens in this period has a long-term impact on their development and behaviour. Kittens handled during this period are more friendly to humans than kittens that did not come into contact with humans [152].

In general, many facilities have developed their own rating systems, but only a few use a specific procedure or guide [153]. These tests have certain limitations; any cat that enters an unfamiliar environment is prone to fear. Even well-socialised cats can show fearful aggression or motivation to escape. As a result, it can be very difficult to initially determine which cats are unsocialised and which have the potential to be adopted or returned to the owner [49]. It was assumed that the stress manifestations of the new environment in socialised cats would present itself differently than the stress manifestations of the new environment as well as people in non-socialised cats. However, it is not possible to separate these two fears [153]. Frightened cats may first begin showing their characteristic behaviour after a few days or weeks when stress levels begin to decline or when they are re-evaluated in a calmer, less stressful environment [76,145]. Slater et al. [49] found that a number of facilities euthanises a cat as soon as it is marked as unsocialised, often on the day of admission to the shelter, without providing time for acclimatisation. In creating an assessment tool for distinguishing between socialised and non-socialised cats, Slater et al. [153] therefore selected a three-day observation period because previous research found that about half of the shelters were able to keep the cat in the facility for at least three days [49] and that some stress reduction can be expected during this period [109]. In shelters where cats are not provided with acclimatisation time, there is an increased probability that socialised animals will be accidentally euthanised and also the likelihood that the original owner will no longer be able to reunite with their animal. The opposite extreme is the situation where unsocialised cat is kept in a shelter for longer than is necessary for the assumption that it will ‘settle’ and prove to be socialised. Keeping non-socialised cats in a shelter for longer than necessary has significant consequences for them in terms of deteriorating living conditions and is not recommended [153,154].

In their approach study, Kessler and Turner [109] described the test they used to assess the degree of socialisation of cats towards other cats. In this test, authors assessed the cat’s reactions to another cat (Cat-Approach-Test). The test was based on a procedure in which a calm, neutered male that was socialised in relation to other cats was placed in a portable box at a distance of one meter from the test subject. Both subjects were allowed visual contact with each other for 1 min. After this time has passed, the behaviour of the tested cat was rated on a scale of 1 (the cat reacts extremely friendly to another individual) to 6 (the cat reacts extremely hostile to other individuals). Cats were considered socialised when they reached an average grade below 3.0 after eight consecutive trials. On the other hand, those individuals who achieved an average mark higher than 4.0 in a series of eight tests were considered unsocialised.

In research studies, rather than exposing a cat to another cat, human approach tests are used. It is generally possible to assess the stress manifestations of cats caused by humans in two main ways; either the test subject is allowed to approach or not approach the experimenter [98] or the experimenter approaches the location of the cat [98,109,155]. A less commonly used method is to hold a cat in one’s arms for a predetermined period of time [156]. The Human-Approach-Test, which was modified by Kessler and Turner [109] on the basis of the Stranger-Approach test [99], the experimenter approaches the cat’s cage from the front, addresses the animal and remains in front of the cage for 1 min while touching the door with their hands. After the time has elapsed, the experimenter opens the door of the housing unit and closes it again. The cat’s response during the test is rated on a 6-point scale (1 = the cat reacts extremely friendly to humans, 6 = the cat reacts extremely hostile to humans). As with the Cat-Approach-Test, the authors suggested how to interpret the results; the cat that received an average grade greater than 4.0 in 8 consecutive trials was considered unsocialised to humans. The cat that received an average grade of 3.0 or less (friendly response to humans) in eight tests was considered socialised. When comparing the results obtained using the Human-Approach-Test and the CSS rating scale, no correlation was found, which raises the question of reliability [121] and validity for both tools. A critical aspect of the tests is the fact that some cats can distinguish between ‘a known and an unknown’ experimenter and adjust their behaviour accordingly, although Horsfield [157] suggests that the difference between cats’ responses to known and unknown individuals is minimal in the tests. The individuality of a cat and socialisation are reported to be the most important factors influencing a cat’s behaviour towards humans [99,149,150,151].

The basic idea of the Human-Approach-Test has been retained in many modifications used across various studies [12,132,133,144,158,159]. One of them is, for example, the Human-Approach Test proposed by Arhant and Troxler [12], who applied the test to cats housed in groups (the original Human-Approach-Test by Kessler and Turner [109] was used only for cats housed individually in cages). The experimenter approached the selected cat in the group in a slow, smooth motion and presented his hand; he stopped if the cat withdrew or when the experimenter’s hand was already 20 cm far from the cat’s head. Direct eye contact with the cat was not maintained during the test. The cat was rated as an ‘animal with which contact is possible’ if the cat did not withdraw and observed the experimenter. Commonly observed reactions included scenting in the direction of the experimenter, sniffing the hand and rubbing the hand. Cats that withdrew during the test, exhibited any form of aggressive behaviour, or were hiding in hiding spots were identified as ‘cats with which contact is not possible’. In groups of 10 cats, all cats in the group were assessed, in larger groups, a sample of 10 cats was randomly selected. If there were cats of similar appearance in the group, a smaller sample of clearly identifiable cats was selected because individuals were not marked.

In contrast to the Human-Approach test by Kessler and Turner, which was used mainly in research studies, the Feline Spectrum Assessment by American Society for the Prevention of Cruelty to Animals (ASPCA) was created for practical use in shelters. The tool consists of 4 evaluation items (greet, hand on cage and cracking the cage door, interactive toy, touch with wand). The animals are tested for three days; the test result is a numerical score that indicates whether the animal is likely to be socialised or not [160]. In addition, to maximise the chance for the cats to show their true natures, the assessment tool should be used four times per cat across three days. It is important that cats are not given a place to hide so they are visible during the assessment items. The disadvantage of the tool is that cats younger than 6 months old and group-housed cats cannot be evaluated. On the other hand, the advantage is that assessment generally takes no more than five minutes per cat to complete, requires a very basic understanding of feline behaviours and its guidelines are available for free on the Internet [160]. For teaching purposes of the evaluators, instructional videos were also created.

The principles of approach tests have been applied to other group of tests, which aim to reveal cats’ personalities. Studies show that many aspects of a cat’s individuality are stable over time [152]; this fact has led and continues to lead to new studies on the temperament of cats [116,161,162,163,164,165,166,167] and on development of tools for assessing the temperament of cats in shelters (Feline Temperament Profile (FTP) [50], ASPCA^®^’s Meet Your Match^®^ Feline-ality™ [168] and its modifications [169,170] and other alternatives of testing [171]). Testing of cats’ personalities contributes to the improvement of welfare as it increases the chance of compatibility between the lifestyle of the new family and temperament of cats, which leads to a reduction in the numbers of cats that are returned to the shelter [50].

### 3.2. Options of Assessing Physiological Indicators of Stress of Shelter Cats

Another category of indicators that can provide insight into welfare of an animal includes physiological indicators, which are mostly used to detect a stress response. A stress response can be assessed either by determining laboratory parameters or by direct assessment of vital signs—most often by measuring blood pressure, heart rate, respiratory rate and rectal temperature. Although these indicators can reliably determine the presence of stress, it seems their applicability in shelters is limited due to several reasons. The analysis of laboratory parameters is often too expensive for the shelter to afford and measurement of changes in vital functions requires specific equipment. In addition, at least minimal handling with an animal is necessary to collect data. This is a significant disadvantage; since measurements are performed to detect stress but handling itself can be stressful to an animal, the results may be skewed and interpreted incorrectly. In order to reduce the handling stress, the animals need to be trained to the measuring procedure; this, however, cannot be applied in shelters. Methods discussed in the following subsections are typically used in research for validating tools, that are easier to use, cost less and are more applicable to shelter settings.

#### 3.2.1. Vital Signs Assessment

Activation of the sympathetic nervous system causes an increase in heart rate, respiratory rate, systolic blood pressure and a decrease in heart rate variability to facilitate the ‘fight or flight’ response [172,173,174]. As was already mentioned in previous sections, cats are extremely sensitive to handling, environmental changes and changes in routine; the result may be a change in physiological values [15]. Changes in physiological values are an established indicator of distress in various welfare studies [172,175,176,177,178] including cats, [173,174,179,180,181,182]; for example, Pratsch et al. [182], used an ear thermometer to monitor body temperature. An increase in body temperature is an accepted indicator of stress [183]. The temperature was measured to detect stress during transport of the cat to the veterinarian; an increase in temperature of 0.5–1 °C was observed, as well as a difference in temperature between the right and left ear, with the temperature of the right ear being higher. The right ear was warmer also in cats with elevated cortisol levels after a visit to the veterinarian, indicating the presence of stress [180]. Although changes in heart rate, respiratory rate and temperature may be sufficient stress indicators, their applicability in the shelters is low as the measures usually require special equipment and skills. Measuring procedures (e.g., wearing a heart rate monitor or taking temperature measurements manually by inserting the thermometer into the rectum or the ear canal) can be a stressful experience for animals, so the handling procedure itself can cause a change in values. Another disadvantage of direct measurements lies in the monitoring of elevations or decreases of values, which, however, is not specific only to stress (but also, for example, to illness or increased activity). Therefore, the assessment of changes in vital signs is possible under conditions where the animal is healthy and does not have enough space to develop intensive locomotive activity, which could cause false elevations of values [98].

#### 3.2.2. Cortisol Assessment

Common methods of assessing the ongoing stress response include assessing hypothalamic-pituitary-adrenal (HPA) axis activity, most commonly by determining cortisol, adrenocorticotropic hormone (ACTH) and glucose [101,180,184,185]. Activation of the HPA axis by a stress stimulus induces the release of ACTH, which increases the synthesis and secretion of cortisol from the adrenal glands [186]. The assessment of physiological indices has an advantage over other evaluation methods in its quantification. The disadvantage is the existence of great individual variability in physiological responses. The levels can also vary over the course of the day—comparing and interpreting the results can, therefore, be difficult [187]. A stress response is complex; a single measurement of a parameter may not reflect subtle changes that occur in the body permanently, which may result in drawing incorrect conclusions about the extent of stress. Factors such as age, animal history, physical individuality and the circumstances under which the samples were obtained must be taken into account when interpreting the levels of physiological indices [188]. Another disadvantage is that the analyses are usually too expensive for shelters, so their use is almost exclusively limited to the scientific context.

Assessment of glucocorticoid levels is a common part of welfare assessment in many animal species especially in a scientific context [189,190,191]. Different types of samples can be used to determine cortisol levels; their aspects in terms of practicality are summarised in a Table 2. For acute stress measurement, it is appropriate to examine blood plasma [192], serum [193] and saliva [50]. Chronic stress, on the other hand, can be detected using non-invasively obtained samples—urine [139], faeces (determination of glucocorticoid metabolites) [164] and fur [194].

In addition to stressors, changes in cortisol concentrations depend on other factors (e.g., sampling and animal handling procedures) [206], which can be a problem in assessing acute stress. The animals should be accustomed to the collection of the necessary sample, otherwise, it is a stressful stimulus that causes a physiological change in the body [207].

The marginal methods used today are identifying the level of cortisol from plasma, serum and saliva. In saliva, cortisol is only found in its free form [198]. Siegford et al. [50] described a collection procedure as a procedure in which the experimenter provided the cat with a cotton swab to chew until it was entirely soaked. For each ‘soaking’, the cat was rewarded with a treat. Pratsch et al. [182] conducted a pilot study aimed at detecting cortisol in saliva—the goal was to investigate the method of saliva collection by cotton swabs and the effects of various food and feeding time on cortisol levels in saliva. All cats were familiarised with sample collection (although the authors do not mention how). Only 23 of the 54 samples contained sufficient saliva (50 μL) for further analysis, so the authors concluded that the method could not be used in the planned study.

Not only saliva collection, but also collection of urine can be problematic as stress in cats can be manifested by urinary retention for the first 24–48 h after admission to the shelter [101,115]. In addition, 25% of all cats admitted to the shelter have blood in their urine [101], which can interfere with the samples meant to be used for analysis. The disadvantage of detecting glucocorticoid metabolites in faeces is the fact that they do not detect short-term stressful experiences. Short-lasting or small fluctuations in the level of circulating glucocorticoids are mostly masked by metabolites accumulating in faeces and bile. Faecal samples containing a detectable amount of metabolites reflecting one-time stress experiences may also be overlooked in the case of irregular sampling [195]. However, unlike some other samples the collection procedure of urine and faeces is relatively simple when using a two-layer cat litter box or a litter box with non-absorbent litter [199] (although with a large number of housed animals sharing toilets, collection from specific individuals may be a bit problematic without constant animal supervision). The analysis of urine and faeces to determine the level of stress has been conducted in various studies focused on cats in shelters. For example, the cortisol to creatinine ratio in urine was found to decrease during the cat’s stay in the shelter [110] and to be significantly lower in cats in enriched shelters than in traditional shelters [101]. An increase in the ratio was found in individually housed cats compared to cats housed in groups [139]. Identifying urinary cortisol levels was also used in a study by Lichtsteiner and Turner [208], who found no difference in the amount of cortisol in cats housed individually and in groups. In similar studies, the same results were obtained by Ramos et al. [165,201], who used faeces as a matrix to analyse the level of cortisol. The level of glucocorticoid metabolites in faeces in cats decreased with increasing time spent in the shelter [202].

Determining the level of cortisol from fur is an innovative method of detection of long-term elevated cortisol in the body [209], first used in 2002 in wild hyraxes (*Procavia capensis*) [203]. Although the process of cortisol incorporation into the hair is not entirely clear [210], in general, it can be said that the free, unbound fraction of cortisol is incorporated into the hair pulp during its growth [211] from blood vessels through passive diffusion [210]. The cortisol-containing sebum secreted by the skin glands is thought to play a role [212]. To determine the presence of stress, the fur must be harvested from previously shaved areas [197] close to the skin and not by plucking to avoid blood contamination [213]. The area and size of the fur collection area vary across studies (in a study by Finkler and Terkel [194], cat fur was collected from a 5 × 5 cm sacral area with small surgical scissors). The advantage of using fur as a matrix is especially the ability to determine the time period in which stress occurred in the animal [204] and the possibility of rapid sampling from a larger number of animals, which is an advantage for animals in group housing. Another advantage is the fact that the stress caused by handling the animals during collection does not affect the concentration of cortisol in the collected fur [205]. Although there was a doubt that the level of cortisol in fur does not represent its actual level in the body but only a kind of ‘local level’ present in hair follicles [214], Stalder and Kirschbaum [215] found indications that hair cortisol reflects the systemic cortisol levels well and seems only slightly affected by the local follicle cortisol production. There was found a connection between the concentration of cortisol in faeces and fur [216] and between its concentration in saliva and fur [189]. However, there is evidence that some factors affect cortisol levels in fur - e.g., colour, which was confirmed in dogs [213]. Debatable factors may be, for example, sex, age, state of health and various areas of the body [204]. Accorsi et al. [216] found a significant correlation between agonistic behaviour and the level of cortisol in the fur, suggesting that a greater amount is found in the fur of aggressive cats. Neutered cats also had lower levels of cortisol in their fur than intact individuals.

Interesting insights can be gained by combining the assessment of behavioural and physiological indicators of cat stress. For example, hiding was negatively correlated with cortisol levels, leading the authors to conclude that this may be important compensatory behaviour in an unpredictable environment [15]. Behavioural assessment is probably a less sensitive indicator of stress than the cortisol-creatinine ratio. The behaviour must, therefore, be extreme for stress to be detectable. Cats with higher cortisol levels have also been found to vocalise more and move less than cats with lower cortisol levels [50]. The spectrum of coping strategies is wide in cats [116]—while some show elements of stress behaviour but have physiologically adapted to new conditions, others have high levels of stress hormones, but altered behaviour is not noticeable.

### 3.3. Options of Evaluation of Observable Health Indicators of Cats in Shelters

The welfare assessment protocol should also include parameters indicating deteriorating animal health [217], including impairment in body condition score [218]. Indicators should be selected to reflect the most frequently observable signs of health problems occurring in cats in shelters. The problems discussed in this section include signs of upper respiratory tract disease, sickness behaviours, gastrointestinal and skin problems, pain in general and changes in body condition. We discuss the methods of evaluating body condition in more details in Section 3.3.2., because only for this indicator more than one validated tool that is well applicable in shelters was created. Efforts to develop valid tools for assessing pain in cats are also intensive, however usability of most of them is low in shelters due to their specificity to the certain context of use or time-consuming procedure. In addition, many of these tools have not been validated yet. In the case of other indicators described in this section, we discuss the reasons why they need to be included in the health evaluation and what results have been obtained by studies that included or studied these indicators.

#### 3.3.1. Health Related Indicators—Signs of upper Respiratory Tract Disease, Sickness Behaviours, Gastrointestinal and Skin Problems and Pain in General

Admission of the cat to the shelter is a stressful experience that can suppress the activity of the immune system and reactivate the subclinical course of the disease (e.g., feline herpesvirus) [5,219,220,221]. Stress can inhibit the production of mucosal antibodies, particularly immunoglobulin A (S-IgA) [222], resulting in increased susceptibility to pathogens causing upper respiratory tract disease (URTD) [223,224,225]. After lack of space, URTDs are the second most common reason for cat euthanasia in shelters in the United States [226,227]. Emotional state and health are interconnected; a decrease in immunoglobulin A (S-IgA) secretion was found in stressed shelter cats [222]. It has, however, been proven that ‘gentling’ (gentle stroking of the head and neck area of the cat together with gentle vocalisation) at regular intervals can help improve the cat’s mental health, increase S-IgA immunoglobulin production and thus reduce susceptibility to upper respiratory tract diseases [16]. Immunoglobin S-IgA is essential for protection against pathogens that may be inhaled or ingested [228] and are highly concentrated in shelters [229]. Higher concentrations of cats are associated with an increase in the prevalence of URTDs [230,231]. Cats that have achieved a high degree of stress according to CSS are almost five times more likely to develop upper respiratory tract infections than cats with low levels of stress [5]. Risk factors in the development of a URTD are the cat’s age (the risk is greater in younger individuals [224,229], the number of days spent in the shelter and specific conditions in the shelter [224]. The development time of a URTD reflects the incubation time of URTD-causing viruses [220,232,233]. Although shelters differ in the prevalence of pathogens, many cats show signs of disease after as little as 1 week spent in the shelter [5,224], according to Dinnage et al. [227] up to a third of the shelter cats suffer from a URTD. Therefore, it is appropriate to include an assessment of the absence or occurrence of eye and nose discharge among health indicators [5]. To evaluate upper respiratory tract infections, Bannasch and Foley [224] developed a three-point scale, the degrees of which express the severity of symptoms (1. absence of upper respiratory tract infection, 2. mild upper respiratory tract infection, 3. moderate to severe upper respiratory tract infection).

In addition to URTDs, as a result of events perceived as aversive by cats, the so-called ‘sickness behaviours’ (SB) occur [113,115]. It is a term denoting a set of non-specific clinical but also behavioural symptoms, which include e.g., vomiting, diarrhoea, refusal of food, lethargy, fever, drowsiness and a general decrease in activity [234]. Stella et al. [115] observed how unpredictable events (presence of dogs, change of caregiver, loud sounds, grouping of new individuals, irregularities in periods of light and dark, temperature fluctuations, transfer of cats) induced by experimenters influenced the occurrence of SB in cats. A negative impact of the events on the animals resulting in a demonstrably increased incidence of SB was confirmed by the authors [115]; the inclusion of SB as an indicator of deteriorated welfare is therefore appropriate. Similarly, for lower urinary tract diseases and idiopathic cystitis in cats, the relationship between the stressor (especially cohabitation with other cats, limited access to the external environment, sharing of feeding bowls and generally frequent changes) and the occurrence of the disease has been demonstrated [235,236,237].

Gastrointestinal problems, vomiting and diarrhoea are often associated with stress [115,238]. It seems that stress can affect the integrity of the intestinal barrier, causing an increase in its permeability and local inflammatory response [239]. A large number of shelter cats suffers from diarrhoea caused by dietary issues, stress or illness (gastrointestinal infection, inflammation or neoplasia [240,241]. In a study by German et al. [242] 11.9% of all cats in the shelter had diarrhoea, in a study by Andersen et al. [243] it was up to half of the cats. Most cats had at least one feline or zoonotic pathogen in their faeces, with the most common pathogens in cats with diarrhoea being feline coronavirus and *Tritrichomonas foetus* [243]. Diarrhoea was most common in kittens and cats housed in a group [242], and a higher prevalence of pathogens was also found in the faeces of group-housed cats [243]. In order to determine the severity of diarrhoea, faecal consistency can be assessed, which was the approach chosen in a study by German et al. [242] based on a 6-point scale (1 = severe diarrhoea, 6 = constipation). Monitoring faecal consistency may be a useful health indicator of welfare, but in group housing, where multiple cats share several toilets, identification of an individual with diarrhoea may be challenging [243]. However, there are some procedures that could be used to mark faeces; in the study by Griffin [244], commercially available concentrated food colourings (known as bakers’ pasters) were used as faecal markers in group-housed cats. Cats consumed food colourings in canned cat food. Colourings served as faecal markers by imparting a distinct colour to each cat’s faeces, allowing identification in the litter box. In another study, to facilitate identification of faecal samples from females housed in pairs, each cat was fed a teaspoon of canned cat food daily with one animal receiving 4 mL green commercial food dye mixed into the food [245]. In addition to dyes, glitter and plastic beads were used to mark faeces in various felids in zoos [246].

The state and condition of the coat can serve as additional health indicators of welfare [247], as they reflect changes in normal grooming behaviour, food quality, the presence of social conflicts and chronic diseases [248,249]. There is a clear link between the skin and the nervous system [250]. Dermatological diseases such as atopic dermatitis can be caused by stress, which is likely to cause itching [251]. Disorders associated with excessive coat care (e.g., psychogenic alopecia) manifest in the form of alopecic areas on the caudal part of the body [126]. It was found that the worsened coat condition of shelter cats correlated with the average length of stay of a cat in a shelter (the longer the cat was in the shelter, the greater the risk of deterioration of coat quality), fewer toilets per cat and unpleasant odour [218]. Similarly, the number of food bowls and their location were associated with a worsened condition of the coat—the monitored shelters, where the study was performed, an average of two bowls was assigned to three cats. The condition of the coat may indirectly indicate the occurrence of conflicts between cats, in which there are fights and, as a result, injuries. The presence of injuries, similar to the condition of the coat, can be included among the indicators of welfare [218].

In a study by Arhant et al. [218], a combination of assessment of health indicators (coat condition, body condition, eye and nose discharge) and behaviours was used to assess the welfare of cats in shelters housed in groups. The authors found that of all the monitored indicators, only two of them were stable over time (body condition and coat quality) as the composition of cats in the shelter was constantly changing. These two indicators correlated with repeated visits to the shelters. Inter-rater reliability was found to be good for coat quality but only moderately good for body condition. To ensure better agreement between observers, the authors recommend improving the evaluation guidelines by providing pictures and more detailed instructions. Authors suggested that body condition and coat quality are feasible and valid for assessing impaired welfare in cat shelters.

To assess the welfare of stray cats and abandoned cats, a scale with five components related to cat health was used in a New Zealand study [252]—body condition score (BCS), coat condition, presence of nasal discharge, presence of injuries and the ear crusting. The authors found no difference in the welfare of free roaming companion cats and managed stray cats. They also suggested further validation of the scale using a more detailed physical examination and a determination of biochemical parameters that would complement the overall picture of welfare.

A problem in evaluating health indicators is that cats generally tend to hide the clinical signs of the disease [89]. Some cats show no signs of pain at all, if they are supervised by humans, other animals, or in stressful situations [253]. The absence of indicators does not necessarily mean that the animal is healthy. With a large number of cats in a shelter, it can also be problematic to notice subtle deviations indicating deterioration in health, especially if the shelter caretaker has only a minimum amount of time to provide care to one animal. In shelters, there are animals with an unknown history; therefore, the caretakers have no way of determining ‘normal behaviour’, that is, the temperament of the given cat, and deviations in behaviour specific to each individual exhibited in case of deterioration. Furthermore, in shelters where cats are housed individually in cage housing with minimal freedom of movement, the caretaker may not notice a reduction in activity or signs of pain, or he may not be able to distinguish an inhibition of activity due to stress from symptoms of an emerging disease [253]. Group housing, on the other hand, is characterised by the placement of several cats in a single room containing equipment providing the cat with a place to hide [140], which may prevent direct visual inspection of shy or frightened individuals. Distinguishing between hiding as a manifestation of pain and hiding as a manifestation of stress can be problematic.

Tools based on animal observation are used in veterinary medicine to assess pain; observation is a non-invasive and effective method that can be used by different people and in different contexts [254]. Several tools for detecting cat pain have been developed [255], but only few are valid and reliable. An example of such a tool is the UNESP-Botucatu scale [256,257]; however, it is a tool applicable only if the cat has undergone an ovariohysterectomy. Although cats are routinely neutered in most shelters, the UNESP-Botucatu scale, with its specificity, focuses only on the detection of pain after this procedure; the limited context of use thus excludes it from the detection of pain in general. However, the Glasgow composite measure pain scale-feline (CMPS-F) is available, which is a validated tool that can be used for assessment in a broader context, specifically in the presence of acute pain in cats [258]. In order to improve its discriminatory ability, it has been updated and its final version includes two additional pain-detecting markers [259].

In addition to assessing behavioural changes, detecting changes in facial expressions also has the potential to indicate the emotional experiences of animals and to provide valuable information about their internal condition. Assessment of facial expressions using the so-called Grimace Scales can be a useful, valid and reliable tool for determining pain [260]. Manifestations are objectively measurable using the ‘facial action coding system’ (FACS) assessing the animal’s facial movements (so-called action units—AU). The system assigns codes to the activity of an individual muscle or group of muscles. In the case of cats, a specific coding scheme (CatFACS) has been developed [261].

Grimace Scales are a simplified method of evaluating facial expressions that can be specifically applied to assess pain. In the case of cats, the Feline Grimace Scale (FGS) was recently developed, which, according to the authors [262], is a valid assessment tool for detecting the presence of pain in cats; to confirm the applicability of the tool in practice, Evangelista et al. [263] applied the scale in assessing pain in cats after an ovariohysterectomy and found good applicability. The methodology was based on the evaluation of facial expressions using images and real-time scoring. The standard methodology for assessing facial expressions of pain using grimace scales is usually based on image scoring [264]; screenshots are obtained from videos, but their analysis is time-consuming. In practice, pain should be assessed immediately and easily [263].

#### 3.3.2. Methods Evaluating Body Condition (Body Condition Scoring, S.H.A.P.E System, Muscle Condition Scoring)

A change in weight can be another indicator of the welfare of cats in shelters. Assessing body condition is a traditional and relatively easily applicable approach to welfare assessment. Previous studies have shown a link between body condition, health, behaviour and welfare [5,265]. There are several methods to describe the appearance of the body and the distribution of body fat, their practical aspects are summarised in a Table 3.

The most commonly used is the semi-quantitative Body Condition Scoring (BCS), which uses visual and tactile assessment of the body. The body condition of the animal is sorted into three categories as underweight, optimal condition and overweight [273]. Areas assessed in cats include the chest, hips, abdomen and waist [274]. A general problem in assessment can occur in long-haired cats when the silhouette of the body is not clearly visible.

A 9-point scale has been well-received—individual levels correlate with the amount of body fat detected by dual-energy X-ray absorptiometry (DXA) [266]. To facilitate the assessment, the assessor may be provided with a series of illustrations of cat silhouettes—these reflect the characteristics of each point of the scale. The method has been validated and is considered reliable; scores determined by independent observers correlate [266]. The disadvantage of the scale is its inability to detect small deviations; therefore, it should not be the only method chosen to monitor changes in body weight [5]. At present, in addition to the 9-point scale, a simplified 5-point model of the BCS system is also used. Its practicality seems to be better—the deviations between the stages are more noticeable, which in practice seems to be an advantage. Each point on the scale represents a 7% increase in body fat. Grade 3–3.5 represents optimal weight with 12 to 19% body fat [269]. 5-point scale has not been validated by DXA. However, good reproducibility was found when comparing results of experienced and inexperienced evaluators [269].

Relatively new approaches to fitness assessment include the S.H.A.P.E. system (Size, Health and Physical Evaluation), which was developed to minimise variability in assessment across observers. It is an algorithm-based system, using the principles of existing scoring systems with a difference in grading—each category of body condition is assigned a letter from A (underweight) to G (obesity). The use of the rating scale does not require previous training of the observer [270], so it could potentially be used by shelter caretakers. As with the 9-point scale, a strong correlation was found between densitometry and the S.H.A.P.E. system [270]. The disadvantage of S.H.A.P.E. and BCS is their relatively small informative value—they do not assess the state of muscle, only the storage of body fat [275].

Musculature can be assessed using the Muscle Condition Scoring (MCS) system; it contains four levels of evaluation—normal musculature, mild, moderate and severe muscle loss [276]. When verifying the applicability of MCS, good repeatability but only moderate reproducibility was found [271]. The animal’s spine, scapulae, skull and wings of ilea are palpated during evaluation [276]. Although the animal may appear to be overweight, it may also suffer from significant muscle loss; in contrast, animals with low a BCS score may show minimal loss of muscle [277]. Overall, the system is mainly used in underweight animals that undergo muscle mass catabolism due to disease or injury [276], and an example of the system’s use is a study monitoring the changes of body condition in cats with hyperthyroidism [278].

The use of direct body weight measurement is an additional option that should be considered in body condition assessment. However, direct measurement, similarly to BCS, does not depict the ratio between individual body components (fat and muscle) [272], so its use is not appropriate when monitoring these components is necessary. In addition, weighing procedure requires the use of equipment. On the other hand, the advantage is that the measurement is easy and quick.

Impaired body condition may predispose a cat to euthanasia [159] or to death in general [279,280]. Body condition assessment can provide useful information related to the health aspect of welfare, as stress is often associated with reduced food intake in cats [5,113,281]. Some cats refuse to eat completely during the first days in the shelter [108]. Weight loss due to anorexia has a serious effect on health because it increases the risk of hepatic steatosis [17]. In a study of 3219 cats in a shelter, 1.5% of cats were found to be underweight (which is equivalent to point 1 on the scale) [282]. In shelter cats, weight loss was observed during the first two weeks spent in the shelter [5] despite providing adequate food; authors of the study suggested the weight loss was probably caused by low food intake associated with stress (food intake and CSS were correlated). However, weight loss may also indicate a serious health problem [283]. In a study by Tanaka et al. [5], 59% of all admitted cats lost their original body weight in the first week in the shelter alone. An association between impaired condition and the development of upper respiratory tract diseases has also been found [5], as well as an association between the level of body condition and the quality of housing [218]. The increased number of underweight cats correlated with housing with less than one resting place per cat, and housing with a lower number of hiding spots. Cats that were given the opportunity to hide were able to adapt faster to the new conditions in the shelter, and their weight loss was not as pronounced as that of cats that did not have this option [108].

## 4. Conclusions

By being placed in a shelter, cats encounter a number of stressful stimuli, which can disrupt the state of their well-being. Although the efforts of facilities to improve the quality of life of cats in shelters have increased significantly in recent years, as the overall interest in welfare issues has increased, it is necessary for experts to come up with new knowledge that would be applicable in practice. Efforts to date have succeeded in enforcing certain measures based on a deeper understanding of the biology and behaviour of cats—for instance, practical improvements such as sound insulation of housing units from dog sounds and other stressful sounds, access to windows and natural light sources, sufficient number of hiding places and elevated locations or placement of housing units above floor level, giving cats a greater sense of security [105,284,285]. The current research also focuses on procedures and methods of management and enrichment of the environment (e.g., playing specific music to cats to relieve stress [286], various forms of olfactory [287,288], cognitive [289,290], visual [291], food [292], pheromone enrichment [293] or social enrichment in the form of human interaction [103]). Another trend in the design of new facilities is group housing of cats, but opinions on it differ in the scientific community. In certain respects, this type of housing may be a form of social enrichment; it has been found that cats from multi-cat households had a higher level of stress after entering a shelter when placed alone than cats from single-cat households [101]. On the other hand, groups of cats may compete for the resources provided; in a study by Dantas—Divers et al. [294], 143 conflicts over 4 days were recorded, of which 44 involved competition over resources.

Given the experiences that cats perceive as aversive in a shelter environment, the need to study appropriate welfare indicators and the development of new tools is immense. The aim of using welfare assessment tools is to reveal critical aspects of living conditions and at the same time establish care management so that disruption occurs as little as possible. As it is not entirely possible to satisfy each individual due to cats’ unique temperament, personality, history and coping strategies and at the same time to combine the personnel, financial and value structure of the shelter, it is necessary to seek compromises.

The disadvantage of evaluating individual indicators separately is the fact that the results provided by the indicators may not match [207]. While behavioural measurements are valuable and commonly used indicators because they can be used immediately, are non-invasive, and require a relatively short time to train the observer [295,296], physiological indicators are accurate, quantifying stress levels [187] and can demonstrate the presence of stress even if there is no external behavioural change. As deterioration of health is associated with welfare impairment, health indicators should also be included in the assessment [217]. Although the assessment of environmental indicators has recently faded into the background and the focus has shifted to animals, they can also help to complete the overall picture of welfare.

Although shelters tend to develop their own welfare assessment procedures, there is no consensus among the indicators that should be part of the tools, let alone the validity and reliability of these procedures [297]. Another fact is that shelters for cats are characterised by many significant differences across countries (e.g., in the number of animals—cats housed in large shelters versus small ones or home refuges; housing—individual versus group housing; composition of animals—mixed shelters with cats and dogs at the same time versus cat-only shelters, value structure—kill versus no-kill policy, but also in the length of stay, adoption programs, level of veterinary care provided, capture programs, etc.); given the various factors influencing welfare, finding a tool that is comprehensive, reliable and at the same time applicable (or that at least some of its parts are applicable) is, therefore, a great challenge. Although such a tool is not currently available, efforts to create it are noticeable [97,218]. The study in which such a tool will be developed will have the potential to significantly help to improve many lives of shelter cats globally.

## Figures and Tables

**Table 1:** Behavioural indicators of good and worsened welfare of cats.

Type of Activity	Behaviour	Signs of Good Welfare	Signs of Poor Welfare	Ref.
general activity	exploration of the surroundings	normal occurrence	reduced occurrence or absence of activity (rarely increased occurrence)	[113,114]
behaviour associated with metabolic processes	feeding	normal occurrence	reduced occurrence or absence of activity	[5,113,114,115]
	drinking	normal occurrence	reduced occurrence or absence of activity	[113,114]
	urination	normal occurrence	reduced occurrence or absence of activity; urination outside of the litter box and instead in other locations of the cage	[114,116]
	defecation	normal occurrence	reduced occurrence or absence of activity	[114]
comfort behaviour	resting	normal occurrence	excessive vigilance	[114,117]
	sleeping	normal occurrence	reduced occurrence or absence of activity; feigned sleep; somnolence	[110,113,114]
	grooming	normal occurrence	over-grooming, self-mutilation or reduced occurrence of grooming	[113,114]
	playing	occurrence of playful behaviour (individual play, play with other cats, objects or people)	reduced occurrence or absence of activity	[114,118,119,120]
social interactions	interactions with people	positive interactions with people (seeking human presence, direct contact, staying in proximity); positive responses to human-initiated interactions	absence of or negative response to a human-initiated interaction, particularly redirected aggression and some forms of affective aggression	[17,114,121]
	interactions with conspecifics	present; positive activities (rubbing, allogrooming, not avoiding contact)	absent or negative activities: hostility, aggression, contact avoidance	[109,113,114,117,121,122]
communication	scratching	normal occurrence	reduced occurrence or absence of activity	[114]
	facial marking	normal occurrence	reduced occurrence or absence of activity	[17]
	urine spraying	normal occurrence	increased occurrence	[17,123,124]
other types of reported activities	compulsive behaviour	absence of compulsive behaviour	presence of compulsive behaviour	[125,126,127]
	hiding	hiding as a normal reaction to fearful stimuli or as a part of playful behaviour	effort to hide	[15,110,114,128]
	vocalisation	normal occurrence	excessive occurrence	[114,129]

**Table 2:** Practicality of various types of samples that can be used to determine cortisol levels.

Sample Type	Type of Stress Detected	Main Advantages	Main Disadvantages	Potential Problems	Ref.
plasma	acute; peak concentrations: 5–15 min after stressor exposure	reflects the actual level of cortisol in the blood	invasive (restraint or sedation of cat is necessary—general anaesthesia or installing a permanent catheter is required); sampling requires skills	sampling procedure as well as diurnal patterns, temperature, activity levels may confound results	[190,192,195]
serum	acute; peak concentrations: 30–180 min after stressor exposure	reflects the actual level of cortisol in the blood	invasive (restraint or sedation of cat is necessary); sampling requires skills	sampling procedure as well as diurnal patterns, temperature, activity levels may confound results	[15,193,195]
saliva	acute; concentration: 2–15% of the total amount of cortisol in the blood	less invasive	prior training of cats on sampling procedure is necessary; a relatively big amount of sample is needed for analysis, which can be a problem to obtain (saliva production is reduced during the stress exposure)	sample is easily contaminated by the intake of food and water or by blood from the oral cavity	[50,182,191,196,197,198]
urine	chronic; concentration: 15% to 18% of the total amount of cortisol in the blood, peak concentrations: 9 ± 3 h after stressor exposure	non-invasive; detection of long-term stress is possible; easy sample collection in single housing; sample collection does not requires skills	in group housing, the sample is difficult to associate with an individual as cats share toilets	diurnal patterns, temperature, activity levels may confound results; sample is often contaminated by blood	[101,110,115,139,199,200]
faeces	chronic; concentration: 80% of the total amount of cortisol in the blood, peak concentrations: 24 ± 4 h after stressor exposure	non-invasive; detection of long-term stress is possible; easy sample collection in single housing; sample collection does not requires skills	in group housing, the sample is difficult to associate with an individual as cats share toilets	diurnal patterns, temperature, activity levels may confound results	[164,195,199,200,201,202]
fur	chronic	non-invasive; easy sample collection (does not requires skills); stability of the sample over time; does not require special transport and storage conditions; ability to determine the time period in which stress occurred in the animal	inability of detecting changes in cortisol levels during short periods of time (hours/days)	cortisol levels may vary depending on some factors—still in research	[194,203,204,205]

**Table 3:** Practicality of methods evaluating body condition of cats.

Scoring System	Usability in Shelters	Validated	Advantages	Disadvantages	Ref.
CSS–9-point scale	yes, if written or graphic instructions are provided	yes	each point correlates with a certain amount of fat, therefore estimation of the amount of fat is possible	noticing deviations between scores may be problematic; evaluation is subjective	[5,266,267,268]
CSS–5-point scale	yes, if written or graphic instructions are provided	no	each point correlates with a certain amount of fat, therefore estimation of the amount of fat is possible; deviations between scores are easily detectable	evaluation is subjective	[267,269]
S.H.A.P.E.	yes—does not require previous training	yes	easy to use	little available knowledge on its use—requires further research	[270]
MCS	questionable—require skills	yes	estimation of overall body composition	evaluation is subjective	[267,271]
direct bodyweight measures	yes	no	easy to measure; indicate weight change	does not quantify body fat and musculature; requires the possession of measuring equipment	[268,269,272]
